# Interventional management for secondary intracranial extension of spontaneous cervical arterial dissection

**DOI:** 10.4103/2152-7806.74092

**Published:** 2010-12-17

**Authors:** Michelle J. Smith, Alejandro Santillan, Alan Segal, Athos Patsalides, Y. Pierre Gobin

**Affiliations:** 1 Division of Interventional Neuroradiology, Department of Neurological Surgery, New York Presbyterian Hospital, Weill Cornell Medical Center, New York, NY; 2 Department of Neurology, New York Presbyterian Hospital, Weill Cornell Medical Center, New York, NY

**Keywords:** Cervical artery dissection, stent, transient ischemic attack

## Abstract

**Background:**

Spontaneous cervical artery dissection (sCAD) is an important etiology of stroke and subarachnoid hemorrhage (SAH) in young patients. Anticoagulation and platelet antiaggregant medications are the treatment of choice, while the indications of endovascular treatment are still to be defined.

**Case Description:**

We report two cases of medically refractory sCAD with intracranial extension treated successfully with multiple intra and extracranial stents. The patients were evaluated at 4 years and 1-year follow-up.

**Conclusion:**

Progressive, spontaneous cervical artery dissection with intracranial extension despite adequate medical therapy is rare and associated with worse prognosis. Given the rapid evolution of interventional technology and techniques, if we are better able to predict the cohort of patients that fail medical management, earlier endovascular therapy may be considered.

## INTRODUCTION

Spontaneous carotid and vertebral artery dissection is an important etiology of stroke and subarachnoid hemorrhage (SAH) in young patients.^16^ It occurs when flowing blood separates the intima and tunica media layers through an intimal tear, subsequently forming a hematoma within the vessel wall. The intimal flap can create luminal stenosis, while a weakened media and adventitia layer can result in pseudoaneurysm formation. The mechanism of stroke may be embolic, hemodynamic or both. Additionally, pulsatile arterial blood entering the tear can continue to extend the dissection intracranially. Trauma is the most common etiology, while spontaneous cervical artery dissection (sCAD) occurs less frequently and is at times associated with fibromuscular dysplasia or rare diseases such as Ehlers–Danlos or Marfan’s syndromes.^6^,^16^,^17^

The incidence of carotid and vertebral artery (VA) dissection in the United States is estimated to be 2.5–3 and 1–1.5 per 100 000, respectively.^4^,^15^,^16^ Although causing only 2% of all ischemic strokes it is a compelling disease entity because it is responsible for up to 25% of strokes in young and middle-age patients.^16^ There is a peak incidence in the fifth decade of life, with women presenting approximately five years earlier.^16^ Carotid artery spontaneous dissection usually presents with nonischemic symptoms such as oculo-sympathetic palsy (partial Horner’s syndrome) and neck pain, and approximately 50% of patients will experience a stroke.^1^,^12^,^16^ The common presenting symptom of posterior circulation spontaneous dissection is posterior neck pain and occipital headache, less frequently pulsatile tinnitus and cervical radiculopathy,^3^ and up to 90% of these patients will have associated ischemia in one or multiple vascular territories.^16^ Intracranial spontaneous dissection is less common than extracranial, but has worse prognosis due to a higher incidence of stroke and SAH. Intracranial dissection resulted in permanent disabling deficits in 44% versus 14% of patients with cervical dissection.^3^,^8^

Controversy exists regarding the management of sCAD. All clinicians agree that either anticoagulant or antiplatelet agents should be administered for the prevention of ischemic events; however, the type and duration of treatment is debatable.^4^,^7^ As the incidence of recurrent ischemic events on appropriate medical treatment is low and overall patient outcomes are favorable, endovascular treatment has been reserved for patients failing medical therapy or demonstrating significant progressive dissection.^9^,^19^ No studies are yet available to predict which subset of patients will fail medical management alone.

In situations where maximum medical treatment does fail, interventional management should be considered. Only a handful of case reports exist describing the successful endovascular management of sCAD with progressive intracranial extension.^2^,^5^,^11^ The present article reports two such cases treated with multiple intra and extracranial stenting.

## CASE REPORTS

### Case 1

### Clinical Presentation

A 49-year-old right-handed male patient with no history of trauma presented to the emergency room with a left pronator drift, hemisensory neglect, and left homonymous hemianopia. Magnetic resonance imaging (MRI) revealed small, punctate infarcts in the right middle cerebral artery (MCA) distribution and magnetic resonance angiography (MRA) revealed minimal stenosis of the distal right cervical internal cerebral artery (ICA) suggestive of dissection. The patient was placed on warfarin and was discharged home with physical therapy. Over the next 3 months, the patient experienced several TIAs and progressive weakness of the left hand. Subsequent MRIs revealed new restricted diffusion in the right MCA territory, MRA showed extension of the right cervical ICA dissection into the cavernous and supraclinoid segments. Secondary to failed medical therapy, stent placement was suggested.

### Intervention

**Figure 1:**
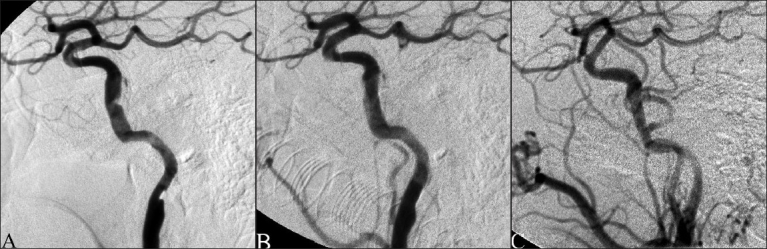
Right internal carotid artery angiogram, lateral view. (A) Preprocedural injection demonstrating dissection of the distal cervical to supraclinoid right ICA. (B) Postprocedural injection demonstrating tandem stents across dissection with no residual stenosis. (C) Eight-month follow-up angiogram demonstrating mild restenosis within the stent

The patient was premedicated with clopidogrel and aspirin. A diagnostic cerebral angiogram with three-dimensional (3D) reconstruction confirmed a long dissection of the right ICA from the high cervical to the supraclinoid segment, with approximately 50% stenosis of the high cervical segment [Figure 1(a)]. The patient was then placed under GETA and anticoagulated with heparin. Over a Transend 14 microwire, five tandem stents were deployed in an overlapping fashion. A Neuroform Treo 4.5 mm × 20 mm self-expanding stent (Boston Scientific, Natick, MA) was deployed in the supraclinoid ICA, 2 Palmaz Genesis 5 mm × 12 mm, and 5 mm × 18 mm balloon-expandable stents (Johnson and Johnson, Miami Lakes, FL) were positioned in the petrous ICA, and 2 Driver 4 mm × 9 mm and 4 mm × 12 mm balloon-expandable stents (Medtronic, Minneapolis, MN) were positioned in the C3 segment of the cavernous and the high cervical ICA, respectively. Postprocedure angiography demonstrated no residual stenosis [Figure 1(b)].

### Postoperative Course

The patient awoke neurologically stable and was discharged home after 3 days. Follow-up angiography, performed at 8 months, demonstrated mild narrowing of the supraclinoid ICA consistent with minimal intimal hyperplasia in the region of the Neuroform stent [Figure 1(c)]. At 22 months, MRI showed no evidence of new infarction and stable encephalomalacia in the right frontal and parietal lobes. On 4-year follow-up examination, the patient’s only residual deficit was mild left hand weakness.

### Case 2

### Clinical Presentation

A 34-year-old right-handed man with a history of hyperuricemia, but no trauma, presented to an outside hospital emergency room complaining of dizziness and stiff neck. While there, he experienced a generalized tonic clonic seizure and was noted to have extensor posturing. He was intubated for airway protection, loaded with dilantin, and hemodynamically stabilized. A computed tomography (CT) scan demonstrated a small area of hemorrhagic conversion of an infarct in the left cingulated gyrus. MRI revealed infarcts in the right dorsal midbrain and occipital lobes and MRA was suspicious for dissection of the bilateral vertebral arteries (VAs). Upon initial exam after transfer, the patient remained in critical condition, only opening eyes to voice, following simple commands, and demonstrating upgaze paresis and left hemiparesis. A noncontrast head CT was stable and CT angiogram demonstrated thrombus in the lacerum segment of the left ICA and stenosis of the bilateral VAs. The patient was placed on a heparin infusion and rheumatology was consulted and performed a workup for vasculitis including serum marker and ophthalmologic examination. All tests were unrevealing.

On hospital day 5 the patient had increasing lethargy and left hemiparesis. A repeat MRI/MRA demonstrated extension of infarctions and dissections. There were multiple new foci of restricted diffusion scattered throughout both cerebellar hemispheres, the subcortical white matter of the left parietal, occipital and frontal lobes, in the left pons and in the lateral left thalamus, consistent with multiple new embolic strokes. There was extension of the right occipital stroke and new restricted diffusion within the right thalamus. The MRA demonstrated increased narrowing of the distal left VA with dissection extending through the entire basilar artery (BA) and occlusion of the right posterior cerebral artery (PCA) as well as extension of the left ICA dissection. Due to failing medical therapy, angiogram and stenting were planned and the patient was loaded with 600 mg clopidogrel and 325 mg of aspirin.

### Intervention

**Figure 2:**
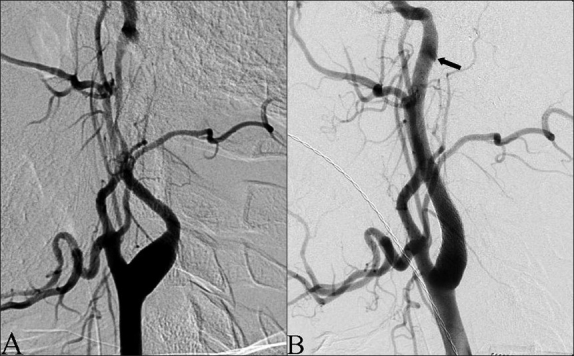
Left common carotid artery angiogram. (A) Preprocedural injection demonstrating significant stenosis of the left ICA secondary to dissection and a pseudoaneurysm of the high cervical segment. (B) Postprocedural injection demonstrating resolution of the stenosis and reduced filling of the pseudoaneurysm (arrow)

**Figure 3:**
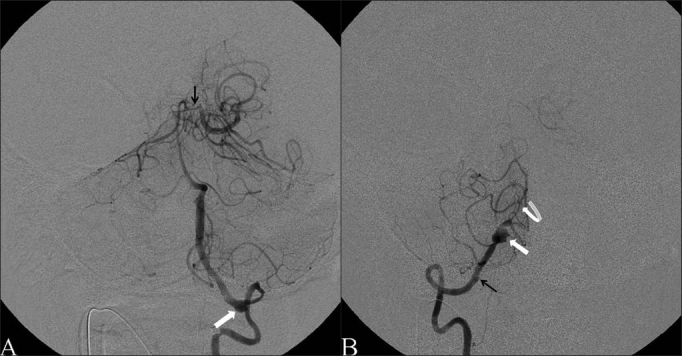
Preprocedural vertebral artery angiogram, AP view. (A) Left VA injection demonstrating dissection and stenosis of the V2–V4 segments of the left VA and BA, occlusion of the P1 segment of the right PCA, severe stenosis of the P1 segment of the left PCA (black arrow), and pseudoaneurysm of the V3 segment of the left VA (white block arrow). (B) Right VA injection demonstrating dissection and stenosis of the V3–V4 segments with small pseudoaneurysm at V3 (black arrow) and large pseudoaneurysm at V4 (white block arrow), with a string sign at the vertebrobasilar junction (white curved arrow)

The patient underwent general endotracheal anesthesia and diagnostic cerebral angiogram revealed dissection and stenosis of the following arteries: cervical left ICA, V2–V4 segments of the left VA, V3–V4 segments of the right VA, basilar artery, and the P1 segment of the left PCA. There was also occlusion of the P1 segment of the right PCA and pseudoaneurysms of the petrous segment of the left ICA, V3 segment of the right VA, and V2–V3 segments of the left VA [Figures 2 and 3, respectively].

**Figure 4:**
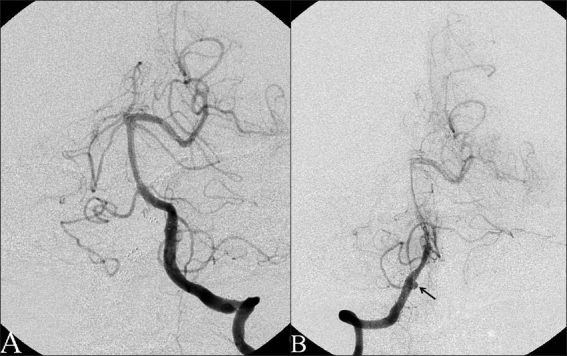
Postprocedural vertebral artery angiogram and fluoroscopy, AP view. (A) Left VA injection demonstrating improved caliber and flow after stenting through the left VA, BA, and PCA, with stable left VA pseudoaneurysm. (B) Right VA injection demonstrating improved caliber and flow through the right VA, BA, and left PCA with minimal residual filling of right VA pseudoaneurysm (arrow)

After systemic anticoagulation with heparin, a Wingspan stent, 4.5 mm × 20 mm (Boston Scientific, Natick, MA), was deployed into the petrous segment of the left ICA. Then, angioplasty of the cervical segment of the left ICA was performed with a Viatrac balloon, 4 mm × 30 mm (Abbott Vascular, Santa Clara, CA) followed by tandem deployment of AccuLink stents, 5 mm × 30 mm and 6–8 mm × 30 mm (Abbott Vascular, Santa Clara, CA). In the posterior circulation, Wingspan stents, 2.5 × 15 mm and 3 × 20 mm, were deployed in the P1 segment of the left PCA and vertebro-basilar segment of the left VA, respectively. Then, the right VA was selectively catheterized and a Wingspan stent, 2.5 × 20 mm, was deployed in the distal V4 segment of the right VA from the basilar junction to the proximal end of the large V4 pseudoaneurysm. Next, a Wingspan stent 3.0 × 9 mm was deployed from the distal end of the large V4 pseudoaneurysm (so this aneurysm would be covered by double layer of stent struts) to the proximal end of the small V3 aneurysm. Finally, because the stenosis of the mid-BA artery persisted, an Enterprise stent, 4.5 × 22 mm (Cordis, Miami Lakes, FL) was deployed in the BA. Postprocedure angiography demonstrated complete resolution of the left ICA stenosis with slight residual filling of the petrous segment pseudoaneurysm [Figure 2(b)]. Additionally, there was significantly improved caliber and flow through the BA and bilateral VAs. The pseudoaneurysms of the V3 and V4 segments of the right VA had only slight residual filling [Figure 4(b)] and of the left VA remained stable [Figure 4(a)].

### Postoperative Course

The patient awoke neurologically stable and was extubated on postoperative day #2. He remained on aspirin 325 mg and clopidogrel 75 mg, continued to neurologically improve and was discharged to an acute rehabilitation center. At 1-month follow-up, the patient demonstrated significant neurological improvement. His only remaining deficits were persistent dysarthria, a left facial droop, and was 4/5 strength in his left upper and lower extremities. He was beginning to walk with a walker and perform activities of daily living.

### Second procedure

**Figure 5:**
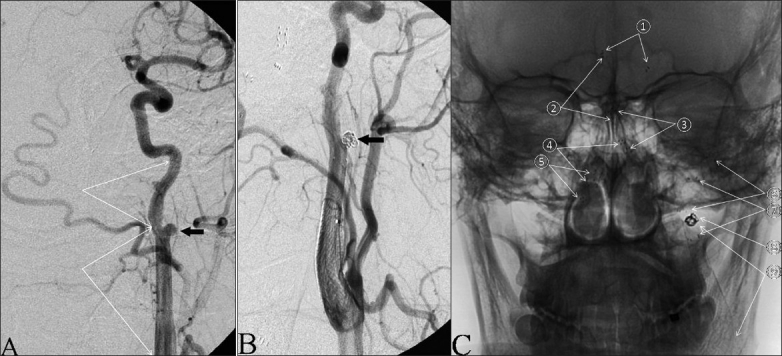
One-month follow-up left CCA angiogram, lateral and AP views. (A) Preprocedural demonstrating a 4 mm pseudoaneurysm (black arrow) of the mid-cervical left ICA between previously stented segments (white arrows). (B) Postprocedural demonstrating minimal residual filling after stent and coil embolization. (C) Unsubtracted fluoroscopy demonstrating previous tandem stents and new coil mass. In the posterior circulation, there are two Wingspan stents across the dissections of the left posterior cerebral artery (1), and left vertebrobasilar junction (3), and an Enterprise stent in the basilar artery (2). The dissecting fusiform right intracranial vertebral artery aneurysm is covered by two Wingspan stents (4) and (5). In the left internal carotid artery, the dissection is covered by a Wingspan stent (6) and an Acculink stent (9), and the dissecting aneurysm is filled with coils (8) and covered by a Wingspan stent (7)

A 1-month follow-up angiogram demonstrated no restenosis of any stented vessel, but showed a new 4 mm pseudoaneurysm of the left ICA in the upper cervical segment [Figure 5(a)]. Of note, this short arterial segment was the only area of the left ICA without previous tandem stents.

Given the rapid development of this aneurysm, decision was made to treat. A Wingspan stent, 4.5 mm × 20 mm, was deployed over the aneurysm neck from within the petrous stent to within the mid-cervical stent and an Axium helix coil, 3 mm × 8 cm, (EV3, Irvine, CA) was used to fill the dome. Postprocedure angiography demonstrated no residual filling of the aneurysm [Figure 5(b) and (c)].

On postoperative day #1 the patient was discharged back to rehab. He has improved further on 1-year clinical follow-up and is awaiting long-term angiographic follow up.

## DISCUSSION

First-line therapy for sCAD consists of antiplatelet or anticoagulant medications because most dissections heal spontaneously and recurrent dissections are only reported in 1%–2% of patients.^4^ Occasionally, however, medical therapy is contraindicated or proves to be refractory, as neurologic symptoms progress despite maximal medical management. During these times, endovascular intervention may be the most appropriate treatment. Patients with critical stenosis or occlusions and limited collateral circulation are at high risk for impending stroke. Additionally, studies have demonstrated that patients with intracranial, dissecting aneurysms presenting with SAH frequently experience fatal re-rupture when left untreated. Therefore, early intervention may be warranted and anticoagulation contraindicated.^5^,^9^,^10^,^13^,^19^

The present two cases were exceptional as they initially had banal cervical artery dissections and were treated with conventional medical therapy. Subsequently, however, they experienced recurrent or worsening ischemic symptoms that were due to intracranial extension of the dissections on multiple segments, leading to numerous severe stenosis and pseudoaneurysms. Multiple stents, five in the first case and nine in the second, had to be placed to restore vascular integrity.

The first patient had progressive, intracranial dissection 3 months following initiation of anticoagulation therapy, diagnosed after symptomatic, mild hand weakness. Although the patient would likely have recovered from his neurologic deficit, repeat imaging demonstrated intracranial extension of the dissection, increasing the risk for future hemorrhage. The second case had stepwise progression of his neurologic deficits, each of which corresponded to extension of his dissection and stenosis. This progression occurred despite being on therapeutic anticoagulation, a set-up for hemorrhagic conversion of his infarctions or pseudoaneurysmal bleeding. The nature of the progression in these patients coupled with examples of successful endovascular intervention in the literature and improved technology and techniques, justified the endovascular treatment they successfully underwent.

A handful of other case reports exist describing stenting as a treatment for refractory spontaneous intracranial artery dissection, all with minimal morbidity and mortality. In a case series by Ansari *et al*., three of nine reported patients underwent endovascular treatment for spontaneous, intracranial dissections. All three presented with SAH and had dissection of the V4 segment of the VA. Two patients underwent stent/coiling and one patient underwent stenting only. Two had complete resolution of stenosis while one had 20% residual. One patient experienced recanalization of an aneurysm requiring retreatment. There were no morbidities or mortalities during intervention or in the postprocedure period. Clinical outcomes were stable or improved for all three patients.^2^

Joo *et al*.^11^ reported a case series of 18 patients who underwent stenting or stent/coiling, nine of which were spontaneous, intracranial dissections of either the supraclinoid ICA(1), VA(7), or BA(1). Two patients failed attempted stent placement. Of the seven remaining patients, one received stent/coil, one received double stent and five received single stent placement. All patients demonstrated resolved stenosis or aneurysm occlusion on follow-up imaging. Only one patient experienced temporary vasospasm treated successfully with intra-arterial papaverine. There were no intra or postprocedural morbidities or mortalities and no patients experienced rebleeding. Biondi *et al*.^5^ described the first patient in the current case series, without long-term follow-up.

These present and previous case series describe overall successful treatment with low morbidity or mortality; however, the interventions are complex requiring experienced operators and special attention to technical considerations. For example, in patients with multiple dissections and pseudoaneurysms, the most flow-limiting vessel should be opened first for several reasons. First, catheters and wires often induce transient spasm within the vasculature. If the most flow-limiting lesion is already opened, this spasm should not have clinical significance. Furthermore, if an adverse event were to occur forcing early termination of the procedure, it would be preferable to have already repaired the most severely affected vessel.

Additionally, one must ensure that only the true lumen is accessed and stented. In the cases described here, gentle injection of contrast through the microcatheter after initial cannulation was used to confirm position in the true lumen. Stenting across a false lumen, especially in the BA which supplies essential perforating arteries, may result in brainstem infarction.

Although medical management will likely continue to be the standard of care for simple sCAD cases given their low rate of ischemic events and high rate of spontaneous recovery, endovascular treatment for complex cases may become more commonplace. The background of reserving interventional therapy as a last resort treatment stems from studies of open, surgical repair reporting relatively high procedure related morbidity,^14^,^18^ and of initial experience with first generation endovascular technology, namely more rigid catheters and wires, and stents with difficult deployment mechanisms.

The collaboration between industry and endovascular surgeons has made new and improved technology available at a rapid pace. In the past few years, longer guide catheters with more compliant tips have become available to safely access the distal cervical and intracranial vessels. Microwires with softer tips and a nickel titanium, or nitinol, core have been developed enabling more directional control, thus reducing the risk of perforating small vessels or aneurysms. Additionally, intracranial stents have been refined resulting in better ability to safely navigate tight turns and more fluid deployment including the Neuroform, Wingspan, and Enterprise stents.

As interventional technology and techniques continue to evolve, and if we are better able to predict the cohort of patients that will fail medical management, earlier endovascular therapy may be considered for sCAD with intracranial extension.
